# Modafinil and abnormal bleeding in a young lady with narcolepsy: a case report

**DOI:** 10.3389/fdsfr.2026.1767644

**Published:** 2026-06-08

**Authors:** Albatol N. Rashed, Ahmed Abdelbasit, Mana Alshahrani

**Affiliations:** 1 Pulmonary Section, Department of Medicine, King Fahad Medical City, Riyadh, Saudi Arabia; 2 Pulmonary Section, Sleep Medicine, Department of Medicine, King Fahad Medical City, Riyadh, Saudi Arabia; 3 Alfaisal University, Riyadh, Saudi Arabia

**Keywords:** abnormal bleeding, hyperprolactinemia, hypersomnia, methylphenidate, modafinil, narcolepsy type 2, pituitary microadenoma

## Abstract

We report the case of a 27-year-old woman with a history of pituitary microadenoma and multiple sclerosis who developed recurrent vaginal bleeding associated with modafinil and methylphenidate use for narcolepsy type 2. The bleeding resolved upon discontinuation of both medications and recurred upon re-exposure. This case highlights a rare but significant adverse effect of stimulant medications and underscores the importance of monitoring in patients with a history of hormonal disorders.

## Introduction

Narcolepsy is one of the most common causes of chronic sleepiness, affecting approximately one in 2000 people (Scammell, 2015). In addition to behavioral changes, first-line treatment typically includes wakefulness-promoting medications such as modafinil (Scammell, 2015). Modafinil acts by competitively binding to the dopamine (DA) transporter on cell membranes and relies on catecholaminergic signaling—both dopaminergic and adrenergic—for its wake-promoting effects (Wisor, 2013). It is generally well tolerated, with headache, nausea, and infections being the most commonly reported side effects, with a lower risk of tolerance compared to methylphenidate (Roth et al., 2007).

## Case report

A 27-year-old woman with a history of multiple sclerosis (MS) diagnosed at the age of 15 presented to the adult sleep medicine clinic with complaints of excessive daytime sleepiness. Her MS had been previously managed with monthly interferon injections, which she had discontinued, and her symptoms had remained stable with no relapses.

At the age of 14, she was diagnosed with a pituitary microadenoma (measuring 6–9 mm) following an episode of vaginal bleeding. At that time, she was found to have hyperprolactinemia, for which she was treated with cabergoline and oral contraceptive pills (OCPs) for 1 year. She discontinued OCPs 2 years prior to the current presentation and had not experienced vaginal bleeding since.

**FIGURE 1:**
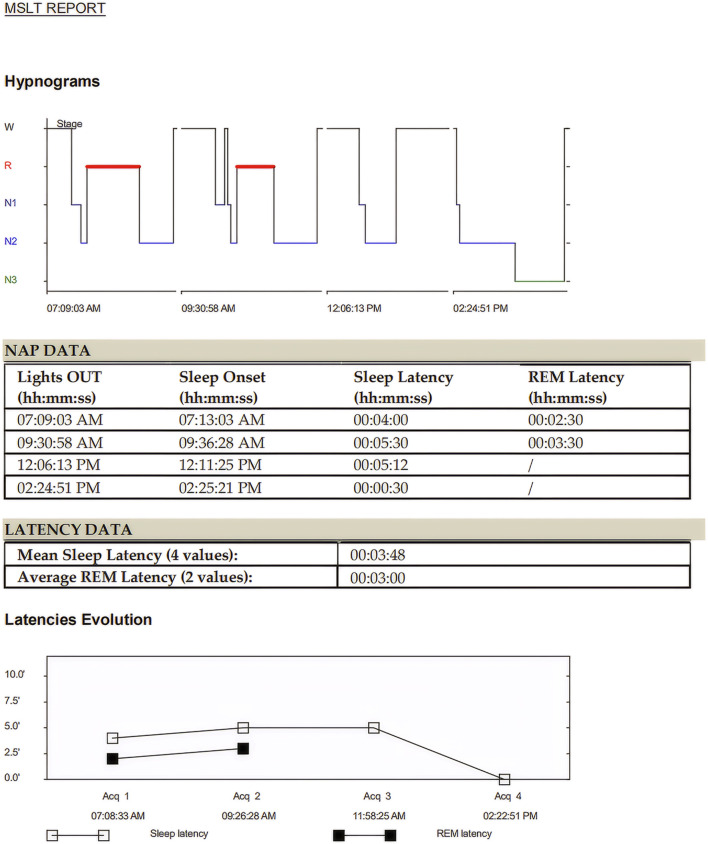
MSLT report sleep latency 12 min 2 cycles of REM seen.

At the sleep clinic, her Epworth Sleepiness Scale (ESS) score was 20 there was no cataplexy, or hallucination, neither sleep paralysis. She underwent overnight polysomnography (NPSG) followed by multiple sleep latency testing (MSLT). The NPSG showed a sleep latency of 12 min, apnea-hypopnea index (AHI) of 0.0, and a sleep efficiency of 90.5%, and Total sleep time 6 h. Her MSLT demonstrated a mean sleep latency of 2 min and 48 s, with two sleep-onset REM periods and an average REM latency of 3 min 00:03:00 (Figure 1). In the absence of cataplexy, these findings were consistent with a diagnosis of narcolepsy type 2.

Modafinil was initiated at a dose of 100 mg twice a day. She noticed vaginal bleeding on the second and third days of using the medication, which coincided with the first day of menstruation and subsequently persisted beyond the usual 7-day duration. The bleeding was mild in amount and with no other bleeding from any other source. She was not on any medication that can cause or worsen the bleeding, such as aspirin or omega-3. Over the upcoming month, the bleeding worsened, for which she received tranexamic acid intravenously and orally for 4 days, but the bleeding did not improve. The decision was taken to stop modafinil, and after 11 days, the bleeding stopped. Given her history of pituitary microadenoma and hyperprolactinemia, a hormonal workup was conducted during the bleeding episode. Her prolactin level was elevated at 40 μg/L. Before initiating modafinil, the prolactin level was 119 μg, thyroid function tests and coagulation profile were within normal limits, testosterone 0.90 ng/dL, follicle-stimulating hormone 4.80 mIU/mL, slightly low luteinizing hormone at a level of 1.70 IU/L, and pelvic ultrasound was unremarkable, and endometrial thickness was within the expected range.

Due to academic demands, the patient resumed modafinil, which again coincided with the start of menstruation, and it triggered vaginal bleeding. The bleeding persisted even after stopping the medication and required treatment in the emergency department and received tranexamic acid IV and orally with no improvement after 3 days. Hemoglobin dropped to 6.6 g/dL, and iron deficiency was noted; platelet level was normal 390 × 10*9/L. She received a blood transfusion and resumed OCPs.

As an alternative therapy for narcolepsy, methylphenidate 18 mg daily was initiated, 10 days before menstruation. Within 4 days, the patient developed spotting that did not progress to vaginal bleeding; the bleeding resolved spontaneously within a few days, while on treatment. Methylphenidate was discontinued after 1 week in accordance with the patient’s preference.

## Discussion

Narcolepsy is a chronic neurological disorder characterized by an irresistible and sudden urge to sleep. Despite its relative frequency, diagnosis is often significantly delayed, typically taking between 5 and 15 years, and up to half of affected individuals may remain undiagnosed (Scammell, 2015). Advances in research have led to the classification of narcolepsy into two types: Type 1 narcolepsy, which is associated with a significant loss of hypothalamic neurons that produce the neuropeptide hypocretin (also known as orexin), and Type 2 narcolepsy, the cause of which remains unclear (Scammell, 2015). The five hallmark symptoms of narcolepsy are excessive daytime sleepiness, cataplexy, sleep paralysis, hypnagogic hallucinations, and disrupted nocturnal sleep. Notably, the presence of cataplexy is strongly linked to hypocretin deficiency (Fronczek et al., 2007).

The American Academy of Sleep Medicine recommends modafinil as a first-line treatment for excessive sleepiness due to narcolepsy, with methylphenidate effective for daytime sleepiness due to narcolepsy (Maski et al., 2021).

The typical starting dose of modafinil is 200 mg once daily, usually in the morning. For patients with persistent sleepiness, a split dosing regimen (e.g., 200 mg in the morning and 200 mg at noon) may be considered to improve late-day wakefulness (Morgenthaler et al., 2007). Serious adverse effects, though rare, can occur and include severe skin reactions such as Stevens-Johnson syndrome and toxic epidermal necrolysis, reported in a 40-year-old woman after 3 weeks of daily modafinil use as a psychostimulant, and histopathology confirmed full-thickness epidermal necrosis with subepidermal blistering, consistent with SJS (Prince et al., 2018). Other serious side effects include aggravating psychotic symptoms in patients with schizophrenia, mania, and hallucinations. It was reported in a 19-year-old lady who was started on modafinil 100 mg to increase her academic performance and presented to the emergency department 5 days later with psychotic symptoms (Aytaş and Yalvaç, 2015). Other serious side effects, although less common, include cardiovascular issues such as chest pain and irregular heartbeats. In a retrospective review of 89 patients with supratherapeutic modafinil exposure, 23 (26.4%) experienced tachycardia, with heart rates ranging from 101 to 168 bpm. Notably, no major adverse cardiac events occurred, and all patients recovered without sequelae (Carstairs et al., 2010).

Currently, there are no documented case reports in the medical literature that directly attribute bleeding to modafinil use. In our patient, a structural, endocrine, and hematologic evaluation was conducted to identify alternative causes of bleeding, but none were found.

This case illustrates a rare and potentially serious adverse reaction to both modafinil and methylphenidate, vaginal bleeding, in a patient with a history of pituitary microadenoma and prior hyperprolactinemia. While the exact mechanism is unclear, dopaminergic and adrenergic stimulation may have interacted with underlying hormonal imbalances, precipitating endometrial instability and bleeding. To our knowledge, this is one of the few reported cases linking wakefulness-promoting agents to significant gynecological bleeding.

## Conclusion

Clinicians should be aware of the potential for vaginal bleeding in patients receiving stimulant medications, especially those with a history of hormonal disorders such as pituitary adenoma. Close monitoring and individualized treatment strategies are essential.

## Data Availability

The datasets presented in this article are not readily available because of ethical and privacy restrictions. Requests to access the datasets should be directed to the corresponding author.

## References

[B1] Aytaş Ö. Yalvaç H. D. (2015). Modafinil-induced psychosis: a case report. Nöro Psikiyatri Arşivi 52 (1), 99–101. 10.5152/npa.2015.7160 28360685 PMC5353011

[B2] Carstairs S. D. Urquhart A. Hoffman J. Clark R. F. Cantrell F. L. (2010). A retrospective review of supratherapeutic modafinil exposures. J. Med. Toxicol. 6, 307–310. 10.1007/s13181-010-0017-6 20358418 PMC2929436

[B3] Fronczek R. van der Zande W. L. van Dijk J. G. Overeem S. Lammers G. J. (2007). Narcolepsy: a new perspective on diagnosis and treatment. Nederl. Tijdschr. Voor Geneeskd. 151 (15), 856–861. 17472116

[B4] Maski K. Trotti L. M. Kotagal S. Robert Auger R. Rowley J. A. Hashmi S. D. (2021). Treatment of central disorders of hypersomnolence: an American academy of sleep medicine clinical practice guideline. J. Clin. Sleep Med. 17 (9), 1881–1893. 10.5664/jcsm.9328 34743789 PMC8636351

[B5] Morgenthaler T. I. Kapur V. K. Brown T. M. Swick T. J. Alessi C. Aurora R. N. (2007). Practice parameters for the treatment of narcolepsy and other hypersomnias of central origin. Sleep 30 (12), 1705–1711. 10.1093/sleep/30.12.1705 18246980 PMC2276123

[B6] Prince V. Philippidou M. Walsh S. Creamer D. (2018). Stevens–johnson syndrome induced by modafinil. Clin. Exp. Dermatology 43 (2), 191–192. 10.1111/ced.13282 29028129

[B7] Roth T. Schwartz J. R. Hirshkowitz M. Erman M. K. Dayno J. M. Arora S. (2007). Evaluation of the safety of modafinil for treatment of excessive sleepiness. J. Clin. Sleep Med. 3 (6), 595–602. 10.5664/jcsm.26970 17993041 PMC2045706

[B8] Scammell T. E. (2015). Narcolepsy. N. Engl. J. Med. 373 (27), 2654–2662. 10.1056/NEJMra1500587 26716917

[B9] Wisor J. (2013). Modafinil as a catecholaminergic agent: empirical evidence and unanswered questions. Front. Neurology 4, 139. 10.3389/fneur.2013.00139 24109471 PMC3791559

